# Supplementary material for: The level of health anxiety before and during the COVID-19 pandemic
Source: PLoS One. 2023 May 24;18(5):e0285799. doi: 10.1371/journal.pone.0285799 (PMC10208469; doi:10.1371/journal.pone.0285799)
Supplement: Attachment — Submitted filename: Response to reviewers.pdf [file pone.0285799.s001.pdf]

**Review submission ID PONE-D-23-01947, entitled "The level of health anxiety before and during the COVID-19 pandemic"**

Dear Dr. Abdu,

We thank you for considering our article for publication in PLOS ONE, and we thank both editor and the reviewers for the constructive and valuable comments. We have considered all the specific recommendations and comments from the reviewers and have corrected and improved our manuscript point-by-point based on their recommendations. Please see our response in the section below.

Best regards, on behalf of the authors,

Anja Davis Norbye

**Editor's comments:**

The article is interesting and may provide important information for public health interventions at local level, but I suggest to compare these results with other experiences that consider aside COVID 19, other cofactors associated to anxiety during the pandemic, especially in European continent (see doi: 10.3390/ijerph191911929). These issues were not included in the analysis but they must be, at least, discussed.

Response from authors:

Thank you for your interest in our manuscript and to point out other challenges evolving in Europe at this present time. We have included a short paragraph in the discussion section (p. 14, l. 276-280) where we discuss how the 21st challenges can affect HA in years to come.

**Comments from reviewer 1:**

Thank you for the opportunity to review "The level of health anxiety before and during the COVID-19 pandemic". In the present study, authors investigated the levels of health anxiety before and during the COVID-19 pandemic in an adult, working population in Norway. They didn't observe any significant alteration in health anxiety scores between the pre-pandemic period and the COVID-19 pandemic period. This paper is well written. I have only minor change to further improve the quality of this paper.

1. Comment from reviewer:

In the introduction, I suggest expanding the topic of HA in relation to COVID-19 with studies on different populations, as this is later discussed as a limitation of the research.

Response from authors:

Thank you for your comment, we agree that this should be addressed although few studies have examined HA in different subpopulations of the general population longitudinally. We have included a sentence on vulnerable populations with regard to HA in page 3, l. 58-59.

2. Comment from reviewer:

I suggest specifying the month of the period referred to in 2019 to understand how broad this period is and to subsequently evaluate the limits of the temporal ranges of the periods under examination.

Response from authors:

Thank you for pointing this out, we have specified the month of 2019 in the methods section (p. 7, l. 127) as well as specifying the time period range in Fig 1. We have also included a short comment regarding the different ranges in time periods in the discussion section; p. 13, l. 252 – 255.

3. Comment from reviewer:

I would suggest adding the implications of the study that are missing.

Response from authors:

We have now added an implications-paragraph on page 14, l. 273-281.

4. comment from reviewer:

I suggest adding in limits paragraph that many variables that may influence health anxiety during the pandemic period were not considered, such as the risk of contracting or having contracted the virus, or having family members who were ill with Covid-19

Response from author:

Thank you for this comment, we have now included a limitation section concerning COVID 19-specific questions on page 12, l. 240-243.

**Comments from reviewer 2**

1. Comment from reviewer:

The manuscript is very interesting and well-written, and the topic is very relevant. However, it is my belief that the paragraph “Statistical analysis” in the section “Material and Methods” should be

clearer, with a more in-depth explanation.

Response from authors:

Thank you for this comment. We have added more details in the description, page 7, l. 136-140.

2. Comment from reviewer:

The authors wrote that there were few participants with two or more measurements (n=353), but it is not specified if this number is representative of the population considered in the study.

Response from authors

The results are the same in both the main analysis and the sensitivity analysis, those with two or more measurements are included in both, no effect of covid on health anxiety. The conclusion would therefore the same even if those with two or more measurements are a highly selected group. We have therefore chosen to not include this information in the manuscript.
